# Ready-to-eat cereal and milk for breakfast compared with no breakfast has a positive acute effect on cognitive function and subjective state in 11–13-year-olds: a school-based, randomised, controlled, parallel groups trial

**DOI:** 10.1007/s00394-021-02506-2

**Published:** 2021-02-20

**Authors:** Katie Adolphus, Alexa Hoyland, Jenny Walton, Frits Quadt, Clare L. Lawton, Louise Dye

**Affiliations:** 1 Human Appetite Research Unit, School of Psychology, University of Leeds, Leeds, LS2 9JT UK; 2 The Kellogg Company, Orange Tower Media City, Salford, Greater Manchester UK; 3 HarvestPlus, International Food Policy Research Institute, 1201 Eye Street NW, Washington, DC 20005 USA; 4 Quadt Consultancy BV, Oostvoorne, The Netherlands

**Keywords:** Breakfast, Cognition, Cognitive function, Adolescents, Randomised controlled trial

## Abstract

**Purpose:**

We tested the acute effect of breakfast (ready-to-eat-cereal [RTEC] and milk) versus (vs.) no breakfast on cognitive function and subjective state in adolescents.

**Methods:**

Healthy adolescents (*n* = 234) aged 11–13 years were recruited to take part in this school-based, acute, randomised, controlled, parallel groups trial with two interventions; Breakfast or No Breakfast. The breakfast intervention consisted of ad libitum intake of RTEC (up to 70 g) with milk (up to 300 ml) administered in a naturalistic school breakfast programme environment. Cognitive function was assessed at baseline and + 70 and + 215 min post-intervention in a group-testing situation, similar to a school classroom context. The CANTAB test battery included: Simple Reaction Time (SRT), 5-Choice Reaction Time (5-CRT), Rapid Visual Information Processing (RVIP), and Paired Associates Learning (PAL; primary outcome). Data collection commenced January 2011 and ended May 2011. This trial was retrospectively registered at www.clinicaltrials.gov as NCT03979027 on 07/06/2019.

**Results:**

A significant effect of the intervention (CMH[1] = 7.29, *p* < 0.01) was found for the number of levels achieved on the PAL task. A significant difference between interventions was found when baseline performance reached level 2 (JT, *z* = 2.58, *p* < 0.01), such that 100% of participants in the breakfast intervention reached the maximum level 4 but only 41.7% of those in the no breakfast intervention reached level 4. A significant baseline*intervention interaction (F[1,202] = 6.95, *p* < 0.01) was found for total errors made on the PAL task, indicating that participants who made above-average errors at baseline reduced the total number of errors made at subsequent test sessions following breakfast consumption whilst those in the no breakfast intervention did not. There was a positive effect of breakfast on reaction time and visual-sustained attention. The results also demonstrated interactions of intervention with baseline cognitive performance, such that breakfast conferred a greater advantage for performance when baseline performance was poorer.

**Conclusion:**

Consuming breakfast has a positive acute effect on cognition in adolescents.

## Introduction

Numerous studies have investigated the effect of breakfast consumption on cognitive function in children and adolescents [1–5]. Children and adolescents have received particular attention for a number of reasons. First, breakfast skipping is common among children and adolescents [6, 7]. Second, breakfast has the potential to improve children’s cognitive function at school, which may benefit learning and academic performance [8, 9]. Additionally, children have a higher brain glucose metabolism compared with adults [10]. Furthermore, children and adolescents are subject to a longer overnight fasting period due to higher sleep demands [11]. Therefore, breakfast consumption may provide energy for the school morning.

To date, four systematic reviews of the effect of breakfast on cognitive function in children and adolescents have been published [12–15]. The findings from acute studies comparing breakfast vs. fasting demonstrate that breakfast consumption has a positive, acute, domain-specific effect on cognition measured within 4 h post-ingestion [12, 13]. However, there is inconsistency among results due to methodological issues, which have precluded firm conclusions. The findings of our systematic reviews [12, 13] informed a recent methodological critique of this literature [16]. Here, we reported the key limitations that have hampered a clear substantiation of the acute effects of breakfast on cognitive function. These include a lack of research on adolescents, few ecologically valid breakfast manipulations or testing environments, small samples, insensitive cognitive tests, and rare concomitant assessment of subjective state [16].

A key limitation in acute studies examining the effects of breakfast on cognition is that the cognitive test choice was not driven by previous evidence showing the task to be sensitive to nutritional manipulations. Moreover, global cognitive function tests, are less likely than domain-specific tests to be sensitive to small, acute, dietary-induced changes in healthy children, less directly related to a specific localised cognitive function and may be more useful if assessed over a longer time period during which global cognitive function might vary [17]. Furthermore, most previous acute studies are laboratory based, with few studies conducted in natural settings such as the school environment alongside the normal school day. Previous studies employing breakfast vs. no breakfast comparisons have used either fixed or ad libitum breakfast interventions, with the majority using the former. Whilst a fixed breakfast intervention reduces the variability in intake within the breakfast intervention, it is less ecologically valid and is unlikely to accurately reflect what the participants might usually consume outside of the study. This approach also assumes that a prescribed portion size is suitable for all participants. However, in a heterogeneous sample of adolescents, there is likely to be a large variation in body weight, growth trajectories, levels of physical activity and therefore, energy requirements. It was also deemed necessary to employ an ad libitum breakfast meal as previous research has suggested that deviation from habitual meal size may adversely affect mood and cognitive function [18, 19]. Hence, benefits to subjective mood state and cognitive performance may be most apparent with test meals that resemble habitual meals.

There is also a lack of research in adolescents. This is important given that skipping breakfast is more prevalent in adolescents than any other age group [7] and breakfast clubs are less prevalent in secondary schools than in primary schools [20]. Therefore, strategies to increase breakfast consumption in the school environment may be required for cognitive benefits, but previous research is scarce in adolescents. Adolescence is one of the greatest periods of growth and change throughout the lifespan. There is a dramatic increase in energy and nutrient requirements which coincides with other factors that may affect adolescents’ dietary choices. These factors include increased independence, a greater need for acceptance by peers, rebellious or non-conformist behaviour, increased time spent out of the home (e.g. for school, extracurricular, social or work activities), changes in sleep patterns, reduced parental control and preoccupation with appearance and body-image. Hence, the cognitive response to breakfast consumption vs. fasting may be different compared with younger children. This study was, therefore, conducted to address the methodological limitations of previous research. The aim of this study was to examine the acute effect of breakfast (ready-to-eat-cereal [RTEC] and milk) vs. no breakfast on cognitive function and subjective state in 11–13-year-old adolescents.

## Methods

### Study design

This study employed an acute, randomised, parallel groups design with two breakfast interventions: breakfast (ad libitum RTEC and milk) or no breakfast. All procedures were conducted in the school environment alongside the normal school day. Data collection commenced January 2011 and ended May 2011. This trial was retrospectively registered at www.clinicaltrials.gov as NCT03979027 on 07/06/2019.

### Participants

The study sample consisted of males and females aged 11–13 years who were recruited to take part in the study from a UK secondary school. This secondary school had approximately 1350 pupils, predominantly of low socioeconomic status (68% eligible for Free School Meals). The inclusion criteria were as follows: aged 11–13 years, willingness to consume RTEC with semi-skimmed cow’s milk during the study, ability to follow verbal and written instructions in English, and normal vision with appropriate corrective lenses if required. The exclusion criteria were as follows: inability to understand the objective of the cognitive tests or carry out the tests, behavioural difficulties or attention disorders, administration of any psychotropic medication in the month prior to testing or during testing, food allergies or intolerances which prevent consumption of RTEC and milk (e.g. coeliac, lactose intolerance), acute illness or feeling unwell within the week prior to testing or during testing, and hearing impairment that precluded the normal use of headphones. A power calculation conducted by an independent statistician estimated that a total of 180 participants (90 participants in each study intervention) was required to detect an effect size of 0.42 (based on the outcome measure “Secondary Memory” reported by Ingwersen et al. [21]) comparing breakfast with no breakfast on the primary outcome (visual-spatial memory performance) with an alpha of 0.05 and 80% power. This “Secondary Memory” outcome measure reflected accuracy scores on immediate and delayed free word recall, delayed word recognition and a visual memory task. Ingwersen et al. [21] sample included 32 participants in each group (total of 64 subjects overall) and found a significant effect on the combined computerised cognitive function tests following consumption of either high glycaemic index or low glycaemic index RTECs. We anticipated that the effect size demonstrated in a smaller sample and comparing breakfast types [21] would be smaller than in a fed vs. fasted comparison and therefore that the present study would be adequately powered. Participants were randomised into breakfast and no breakfast interventions. The randomisation procedure was carried out by the independent statistician prior to screening and revealed to the researchers via an excel file after the participant screening sessions. The selected participants for the study were stratified by class and gender. For each stratum, the interventions were randomly assigned, such that half of the participants were assigned to the breakfast intervention and the other half to no breakfast intervention within each stratum. Hence, the trial was balanced for intervention comparison and unbiased with respect to class and gender.

### Intervention

There were two interventions in this parallel groups study:

1. Breakfast: Ad libitum RTEC (up to 70 g), from a choice of four commercially available RTECs with 1.8% fat cow’s milk (up to 300 mls). Ad libitum water intake was also permitted. The four RTECs were corn flakes, toasted rice, shredded whole wheat pieces with a sugar topping, and wheat, corn and oat shapes (Kellogg’s Corn Flakes, Kellogg’s Rice Krispies, Kellogg’s Mini Max, and Kellogg’s Start respectively)
2. No breakfast: Ad libitum water intake.

Nutrient composition of the test breakfasts (per maximal portion) is shown in Table 1. On the test day, participants arrived at school in a fasted state having been asked not to consume any food or drink after 2100 h on the previous evening (with the exception of ad libitum water intake). Breakfast was administered in the school dining area within a typical school breakfast programme environment. Breakfast preparation and instructions to participants were standardised. The RTECs were presented in small, individual plain (unbranded) white boxes in 70 g maximal amounts to each participant. Providing a maximal 70 g portion allowed participants to self-serve and consume a breakfast suitable for them in terms of portion size, and therefore may better reflect their habitual intake vs. a standardised portion size. Milk was served in small, individual glass jugs in 300 ml maximal amounts to each participant. Participants were permitted to self-serve their chosen RTEC and milk in an amount habitual for them and were instructed to eat until they were comfortably full. Participants were required to eat/drink all of the breakfast/water within 15 min. Participants in both interventions were permitted ad libitum water intake during the 15 min breakfast session. This intervention was chosen to closely resemble a typical school breakfast context and composition. Participants completed a self-report written questionnaire at the screening. The questionnaire contained three items relating to the participant’s habitual breakfast consumption frequency and food type.

**Table 1:** Nutrient composition per maximal portion of the test breakfasts

	Corn flakes^a^ (Kellogg’s Corn Flakes)	Toasted rice^a^ (Kellogg’s Rice Krispies)	Shredded whole wheat sugar topped pieces^a^ (Kellogg’s Mini Max)	Wheat, corn and oat shapes^a^ (Kellogg’s Start)	Milk^b^
Energy (kJ)	1123	1138	1096	1154	618
Energy (kcal)	265	268	259	273	147
Protein (g)	4.9	4.2	7.7	5.6	10.2
Total carbohydrate (g)	58.8	60.9	51.1	55.3	15
Sugars (g)	5.6	7.0	12.6	16.8	15
Total fat (g)	0.6	0.7	1.4	2.5	5.1
Saturated fat (g)	0.1	0.1	0.2	1.4	3
Fibre (g)	2.1	0.7	5.6	3.5	0
Salt (g)	0.9	0.8	0.0	0.7	0.18

^a^Maximal portion size: 70 g; nutrition information was provided by the manufacturer (Kellogg’s)

^b^Maximal portion size: 300 ml; nutrition information was provided by the manufacturer (Sainsbury’s)

Following the breakfast session, the amount of RTEC and milk leftover was weighed and recorded to determine the amount consumed. Throughout the remainder of the morning, participants were permitted ad libitum water intake only until the school’s scheduled lunch period. The school had a policy that pupils were not permitted to eat or drink (except water) during lessons which aided compliance with the fasting regime.

### Cognitive function test battery

The Cambridge Neuropsychological Test Automated Battery (CANTAB; Cambridge Cognition Ltd) was used to assess cognitive function. The battery was administered on individual touchscreen portable computers. Testing was conducted in groups of 15–20 participants, in a quiet classroom which was consistent across test days. Cognitive testing was conducted in a group-testing situation to closely resemble a typical school classroom context. Standardised administration scripts were used to ensure consistency in administration. The 25-min cognitive test battery comprised four tests administered in the following order: Simple Reaction Time (SRT), 5-Choice Reaction Time (5-CRT), Rapid Visual Information Processing task (RVIP), and Paired Associates Learning (PAL). The cognitive tests employed had demonstrated sensitivity to similar acute nutritional manipulations in previous studies [21–25]. These tests were grouped into three cognitive constructs of reaction time, visual-sustained attention, and visual-spatial memory respectively. Visual-spatial memory (PAL performance) was the primary outcome. This was selected as a primary outcome as previous studies have demonstrated that visual-spatial memory is a predictor of academic performance, including children’s reading and mathematics skills [26–28].

### Primary outcome: visual-spatial memory task

The PAL task was employed to measure immediate visual-spatial memory. The duration of the task is typically 7–9 min, depending on response times and level reached. The task consists of one practice level followed by four assessed levels. At each level, white boxes are displayed on the screen and these open in a random order. Depending on the level, two or more of these boxes contain patterns. After all boxes have opened, each previously presented pattern is shown in the centre of the screen and the participant is required to indicate the previously shown location of the pattern by touching the relevant white box on the screen. As the task proceeds, these assessed levels increase in difficulty by increasing the number of patterns presented. The number of patterns presented at levels 1, 2, 3 and 4 are 2, 3, 6 and 8, respectively. At each level, the participant is given a maximum of six attempts (termed “trials”) to recall all of the correct pattern locations. If a participant is unable to recall all of the correct pattern locations within six attempts, the test terminates. Hence, a participant has to succeed at one level to advance to the next level. Parallel forms were presented at each test session. Outcome variables for this task were errors at each level, total errors (adjusted), trials at each level, total trials (adjusted), correct responses on the first trial within each level, and levels achieved. The total errors and total trials outcome variables are adjusted scores. The total trials (adjusted) variable refers to the number of trials attempted throughout the entire task. Some participants did not reach level 4 (8 patterns) because they did not complete level 3 (6 patterns). Hence, the total trials score is adjusted for levels that they did not reach (it includes an estimate of the number of trials they would have attempted on any levels they did not reach). The total errors (adjusted) variable refers to the number of errors made throughout the entire task with an adjustment for any levels that were not reached, as per the total trials (adjusted) outcome variable.

### Secondary outcomes: cognitive function

#### Reaction time tasks

The SRT and 5-CRT tasks were used to assess reaction time. The SRT task requires the participant to respond to a stimulus (yellow dot within a white circle) presented in the centre of the computer screen by touching the screen within 500 ms. The 5-CRT task employs the same paradigm as the SRT task, except the stimulus appears in one of five locations on the computer screen requiring the participant to choose the correct location. Stimulus onset time varied from 750 to 2250 ms. Both tasks involve practice (five trials) and assessed phases (14 trials). Each task lasts approximately two minutes. Outcome variables on this task were decision time, movement time, errors of inaccuracy (response was not within the physical boundaries of the target stimuli), errors of no response (failure to respond within 500 ms), premature errors (response is made before the target stimulus is presented), and total errors (sum of all errors).

#### Visual-sustained attention task

The RVIP task was used to measure visual-sustained attention. Participants are required to detect a 3-digit target sequence within a continuous, rapidly presented digit series on the computer screen within 1700 ms. Participants respond by pressing a press pad upon detection of the consecutive target sequence “3–5–7”. The task consists of a 2 min practice phase followed by a 7 min assessed phase. The first minute of the assessed stage is a ‘run-in’ period; therefore responses from the last 6 min are included as outcome variables. These 6 min (termed blocks 1–6) contain nine target sequences each (54 in total). Outcome variables for this task were correct targets by block (blocks 1–6), total correct targets, false alarms, correct rejections, reaction time, and guessing tendency (A Prime [A′]; B Double Prime [B″])[29].

### Secondary outcomes: subjective state and subjective cognitive test performance

Subjective state was a secondary outcome measure. Concomitant ratings of subjective hunger, cheerfulness, energy, distractibility, ease of focus, bad temper, keenness to try hard, and feeling awake were taken throughout the test morning using 8 unipolar Visual Analogue Scales (VAS). The VAS descriptors were chosen and adapted from those used in previous studies [13] to reflect dimensions of motivation, alertness and mood. The mood descriptors were piloted in a small sample of 11-year-olds to ensure suitability for the study population. VAS were presented electronically using the CANTAB equipment and processed by Cambridge Cognition Ltd. Participants responded to each VAS using the touchscreen by moving the cursor along a 100 mm line with extreme anchors at each end. The initial location of the cursor was at the 50 mm mark. There were 100 points on the scale, yielding possible scores of 0–100. Participants were asked to rate their subjective state immediately before and after breakfast and each cognitive test battery. At each measurement point, participants completed a total of 8 or 12 VAS items. The 8-item VAS (pre-cognitive testing and following breakfast) assessed hunger, cheerfulness, energy, distractibility, ease of focus, bad temper, keenness to try hard, and feeling awake and the 12-item VAS (post cognitive testing only) contained an additional four items relating to perceived test battery difficulty and perceived performance, concentration and frustration during the test battery. The 8-item VAS took approximately 3 min to complete and the 12-item VAS took approximately 4 min to complete.

### Procedure

Participants attended two screening sessions in the week prior to the scheduled test day. At the first screening session, participants completed a self-report written questionnaire to obtain information on habitual breakfast intake, medical conditions, food allergies and intolerances. The height and weight of each participant were measured and recorded by trained researchers to determine Body Mass Index standard deviation scores (BMI SDS) based on the British 1990 growth reference data [30]. Participants were also tested for colour vision. Lastly, participants were given the opportunity to try a small amount of each RTEC (with milk) and choose the RTEC they wished to consume as a test breakfast. Additionally, the following demographic measures were taken from school records: age, gender, ethnicity, and Cognitive Abilities Test (CAT) score. The CAT is carried out routinely by UK schools at the beginning of year 7 and 8. The CAT has three timed, multiple-choice test batteries which yields scores for verbal, nonverbal, and quantitative reasoning ability [31]. A mean CAT score was calculated as the average of the three subtest scores. Mean CAT score was used as an indication of the cognitive abilities of the sample and is a proxy for Intelligence Quotient (IQ).

The full test day schedule and concomitant school activity are given in Table 2. Three cognitive and subjective state testing batteries were administered on the test day. The baseline battery was administered at 0840 h (− 25 min pre-intervention). At 0905 h, participants were served breakfast or no breakfast in the school dining area with 15 min allowed for consumption. The second battery was administered at 1015 h (+ 70 min post-intervention). The third battery was conducted in the late-morning at 1240 h (+ 215 min post-intervention).

**Table 2:** Test day schedule

Time	Time relative to the intervention	Activity	Concomitant school activity
0835	− 30	Registration and arrival at testing classroom	Lesson 1
0840	− 25	Baseline measures VAS 8-item (T1) Baseline cognitive test batteryVAS 12-item (T2)	Lesson 1
0905	0	Study intervention	Lesson 1
0920	+ 15	Post-intervention measures VAS 8-item (T3)	Lesson 1
1015	+ 70	Test session 1 measures VAS 8-item (T4) Test session 1 cognitive test battery VAS 12-item (T5)	Lesson 2 (ends at 10:30) Break-time (10:30 -10:45)
1240	+ 215	Test session 2 measures VAS 8-item (T6) Test session 2 cognitive test battery VAS 12-item (T7)	Lesson 4 (ends at 12:45) School lunch period
1310	+ 245	End of test day	School lunch period

*T* time point, *VAS* visual analogue scale

### Ethical considerations

Prior to commencement of the study, ethical approval was obtained from the School of Psychology Research Ethics Committee at the University of Leeds, UK (Reference: 10-0105, Date: 27/12/2010). All researchers involved in the study obtained Disclosure and Barring Service clearance. To recruit participants, letters were sent home to each parent/guardian of school pupils aged 11–13 years, containing a cover letter and information sheet for the parent/guardian and an information sheet for the school pupils. The pupil version was specifically designed, in terms of readability and content, to aid understanding. For the pupils, this information was reiterated at screening and they were given the opportunity to ask questions. Participants and their parents/guardians were told that participants could withdraw at any point before or during the study without giving a reason. The tasks were not expected to induce any pupil distress and there were no adverse events related to pupil participation. Informed consent was obtained from parents using passive consent (opt-out), and each child gave his/her own verbal assent to participate in the study at screening (opt-in) after reading the pupil information sheet and a presentation on the study from the researchers. Parents/guardians were informed that if they were happy for their child to take part in the study they did not need to respond to the letter or notify the researchers, and consent would be assumed but children opted in on the day and had the opportunity to withdraw at any time. Participants and their parents did not receive payment or another reward for taking part in the research. However, the participants received a certificate at the end of the research to thank them for taking part.

### Statistical analysis

All analyses were performed using SAS version 9.2 (SAS Institute). Data for which residuals illustrated a skewed distribution were normalized by transformation of the data (logarithm of the data) and/or the removal of outliers (where the studentised residual > 3). Baseline participant characteristics were compared using independent groups *t* tests for continuous variables and Pearson’s chi-squared (*χ*^2^) tests for categorical variables.

Cognitive function data that complied with parametric assumptions were analysed using mixed ANCOVA models with the intervention (2 levels; breakfast and no breakfast) as the between-subject factor and session (2 levels; test session one and test session two) as the repeated measures factor with baseline cognitive test performance included as a varying covariate. All main effects and their interactions (baseline*intervention; baseline*session; intervention*session; baseline*intervention*session) were requested in the first model, and all covariates including age, gender, school year, school class, habitual breakfast intake, and CAT score. The model fit, *F* values and significance of main effects and interactions were examined in each model. Non-significant interactions and covariates were removed, starting with the highest order interactions, and the resulting model was compared to the previous model using the McQuarrie Tsai corrected Akaike Information Criterion (AICc) [32]. The AICc gives an indication of the amount of remaining unexplained variance after the model has been fitted, in which a smaller AICc value indicates a better model. Models were chosen on the basis of ‘best fit’, and interaction terms that improved the fit were retained. The reported ANCOVAs are the best fit (i.e., lowest AICc) models. In ANCOVA models, the main effects are a test of the difference at the intercept, where baseline is equal to zero. Therefore, these main effects are informative only when there are no interactions with baseline. As the ANCOVA included baseline as a continuous covariate, the test for significant differences by the intervention was based on the Least Square Means (LSMeans). Where a baseline*intervention interaction was present, the LSMeans test indicated the magnitude of the difference between the two interventions at different levels of baseline. Significant baseline*intervention interactions were also explored by a scatterplot of baseline on post-intervention cognitive performance according to intervention for each outcome as required.

Cognitive function data that did not satisfy parametric assumptions were subjected to the Poisson dispersion test. A non-significant test indicates that a Poisson distribution is adequate and the mean events occur at a constant rate in a typical Poisson distribution. Where the Poisson dispersion test returned a significant result, indicating the absence of a Poisson distribution, the Cochran-Mantel–Haenszel (CMH) test was used as a non-parametric equivalent of ANCOVA with baseline as a covariate. Where a significant CMH test was coupled with a baseline*intervention interaction, the baseline response at which the difference between interventions was statistically significant was determined using the Jonckheere-Terpstra (JT) test [33, 34].

All VAS data were analysed using mixed ANCOVA models with intervention (2 levels; breakfast and no breakfast) as the between-subject factor and the time point (*T*) of measurement (see Table 2) as the repeated measures factor (6 levels; T2–T7 for the 8-item VAS ratings or 2 levels; T5 and T7 for VAS ratings from the additional four items in the 12-item VAS) and baseline VAS ratings as the covariate. Age, gender, school year and school class were included as covariates. The LSMeans test was employed between the interventions at each of the time points to indicate the magnitude of the difference between the two interventions at each post-intervention time point.

## Results

### Participant characteristics

**Fig. 1:**
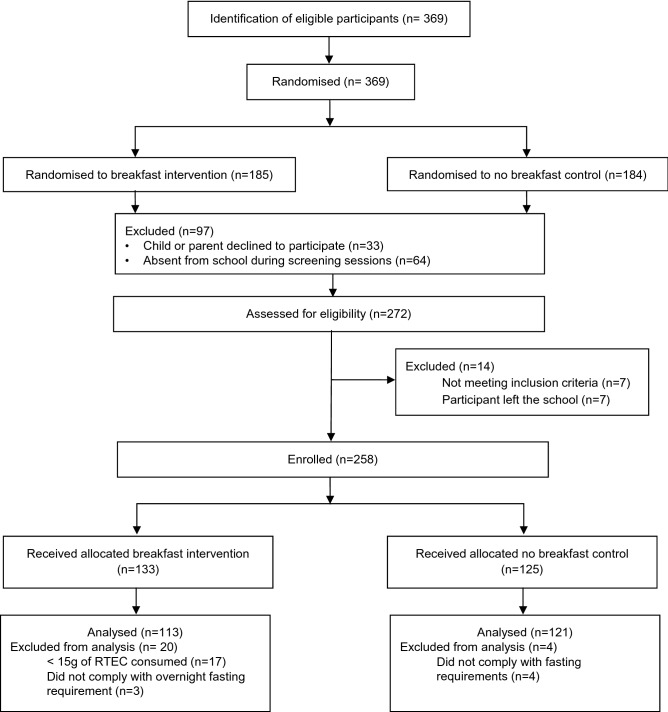
Participant flow chart. RTEC, ready to eat cereal

Flow of participants through the phases of the study is shown in Fig. 1. A total of 369 school pupils were invited to take part, of which a total of 111 pupils were excluded (see Fig. 1). Of the 258 participants enrolled, 24 were excluded from the analysis due to lack of compliance on the test day. This gave a final sample size of 234 participants of which 113 were randomly allocated to the breakfast intervention and 121 to the no breakfast intervention. Hence, this sample provided adequate power (80% power with an alpha of 0.05) based on the power calculation reported in “Participants”, which suggested 180 participants were required. Participant characteristics according to intervention are shown in Table 3. There were no significant differences between the characteristics of participants assigned to each study intervention. The sample consisted of habitual breakfast consumers and non-breakfast consumers, such that 42.7% of participants reported that they consumed breakfast every day (7/days a week) or nearly every day (5–6 days/week), 23.5% of participants reported that they consumed breakfast occasionally (3–4 days/week) and 33.8% of participants reported that they rarely consumed breakfast (0–2 days/week). RTECs were the most frequently consumed food for breakfast on school days (42.9%). Hence, it is likely that the breakfast food provided in the breakfast intervention broadly reflected habitual breakfast intake and was, therefore, ecologically valid.

**Table 3:** Participant characteristics according to intervention^a^

	All participants (*n* = 234)	Breakfast intervention (*n* = 113)	No breakfast intervention (*n* = 121)
Gender, *n* (%)			
M	125 (53.4)	64 (52.9)	61 (54.0)
F	109 (46.6	57 (47.1)	52 (46.0)
Ethnicity, *n* (%)			
White British	159 (67.9)	79 (65.3)	80 (70.8)
Asian/ British Asian	47 (20.1)	29 (24.0)	18 (15.9)
Black British/African/Caribbean	15 (6.4)	8 (6.6)	7 (6.2)
Mixed	8 (3.4)	3 (2.5)	5 (4.4)
Other	3 (1.3)	1 (0.8)	2 (1.8)
Missing data	2 (0.9)	1 (0.8)	1 (0.9)
Habitual breakfast consumption—frequency/week, *n* (%)			
0	18 (7.7)	10 (8.3)	8 (7.1)
1–2	61 (26.1)	23 (19.0)	38 (33.6)
3–4	55 (23.5)	32 (26.4)	23 (20.4)
5–6	38 (16.2)	22 (18.2)	16 (14.2)
7	62 (26.5)	34 (28.1)	28 (24.8)
Age, years	12.43 ± 0.04	12.45 ± 0.05	12.42 ± 0.05
BMI SDS	0.69 ± 0.08	0.60 ± 0.11	0.78 ± 0.12
CAT SAS score	90.51 ± 0.72	90.53 ± 0.98	90.48 ± 1.07

*CAT* Cognitive Abilities Test, *SAS* Standard Age Score, *SDS* Standard Deviation Score

^a^Values are means ± SEs unless otherwise indicated

### RTEC choice and self-serve RTEC intake

Within the breakfast intervention, eight (7.1%) participants chose to consume Kellogg’s Corn Flakes, 12 (10.6%) participants chose to consume Kellogg’s Rice Krispies, 30 (26.5%) participants chose to consume Kellogg’s Mini Max, and 63 (55.8%) participants chose to consume Kellogg’s Start. Across all four RTEC types, total mean RTEC intake was 49.5 g ± 17.6 g. Total mean intake of milk was 133.5 g ± 79.4 g. The overall mean intake of energy was 1059 ± 359 kJ. Overall macronutrient intake was: 44.5 g ± 15.0 g total carbohydrate, 16.0 g ± 6.0 g of which sugars, 9.0 g ± 3.8 g protein, 3.7 g ± 1.6 g total fat, and 2.7 g ± 1.6 g fibre.

### Cognitive function: primary outcome

#### Visual-spatial memory

**Fig. 2:**
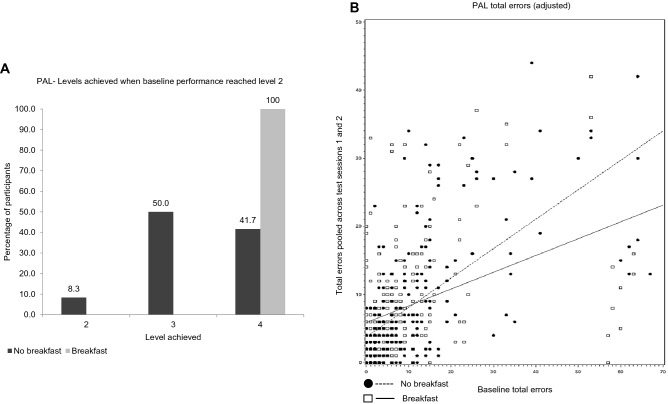
**a** Number of levels achieved on the PAL task according to intervention pooled across test sessions one and two. Figure shows percentage of participants reaching each level when baseline performance reached level 2 only. **b** Scatterplot of baseline performance against post-intervention PAL task total number of errors (adjusted) pooled across test sessions one and two by intervention

A significant effect of the intervention (CMH[1] = 7.29, *p* < 0.01) was found for the number of levels achieved on the PAL task (i.e. the number of levels successfully passed by a participant). Further analysis using the JT test showed a significant difference between interventions when baseline performance reached level 2 (JT, *z* = 2.58, *p* < 0.01) with no significant difference when baseline performance reached level 3 or 4. Figure 2a demonstrates that for participants with baseline performance at level 2, 100% of participants in the breakfast intervention reached level 4 but only 41.7% of those in the no breakfast intervention reached the maximum level 4. Hence, more of those participants who performed poorly at baseline (i.e. those who reached a low level on the task at baseline) improved their performance across the morning following breakfast consumption relative to fasting.

For the total errors made on the PAL test, the distribution of residuals showed a positive skew and was normalised by the removal of eight outliers. The analysis showed a significant baseline*intervention interaction (*F*[1,202] = 6.95, *p* < 0.01) for total errors made on the PAL test. The LSMeans comparison showed no difference between interventions when baseline = 10 (*t*[202] = − 0.25 ns; Table 4) and when baseline = 0 (*t*[202] = − 1.85 ns). However, the LSMeans comparison between interventions when baseline = 50 was significant (*t*[202] = − 2.43, *p* < 0.05). Figure 2b shows a scatterplot of baseline total errors against total errors pooled across test session one and two according to breakfast intervention. Participants who made above-average errors at baseline reduced the total number of errors made at subsequent test sessions following breakfast consumption whilst those in the no breakfast intervention did not (Fig. 2b). There were no significant effects of the intervention on all other PAL outcome variables.

**Table 4:** Cognitive function data that complied with parametric assumptions by intervention and test session

Cognitive function outcome variable	Baseline^a^		TS1		TS2	
	Breakfast	No Breakfast	Breakfast	No Breakfast	Breakfast	No Breakfast
PAL total errors adjusted	9.32 ± 1.15	9.78 ± 1.14	8.27 ± 0.72	8.63 ± 0.68	8.51 ± 0.73	7.88 ± 0.68
5-CRT movement time (ms)	279.15 ± 5.52	292.69 ± 7.29	277.35 ± 4.58	290.68 ± 4.20	274.04 ± 4.62	287.85 ± 4.32
RVP correct targets block 3	7.43 ± 0.16	7.47 ± 0.14	7.17 ± 0.15	7.11 ± 0.14	6.97 ± 0.15	7.03 ± 0.14
RVP correct targets block 4	7.37 ± 0.15	7.50 ± 0.15	7.04 ± 0.14	7.26 ± 0.13	6.95 ± 0.14	7.07 ± 0.13
RVP false alarms	5.99 ± 0.65	5.46 ± 0.61	7.22 ± 0.52	7.21 ± 0.50	8.10 ± 0.54	8.11 ± 0.50
RVP B″	0.83 ± 0.13	0.84 ± 0.13	0.83 ± 0.01	0.81 ± 0.01	0.80 ± 0.01	0.79 ± 0.01

Values are LSmeans ± SEs unless otherwise indicated

*B″* B double prime, *TS* test session

^a^Values are means ± SEs. Baseline cognitive test performance was included as a covariate in the analysis

### Cognitive function: secondary outcomes

#### Reaction time

**Fig. 3:**
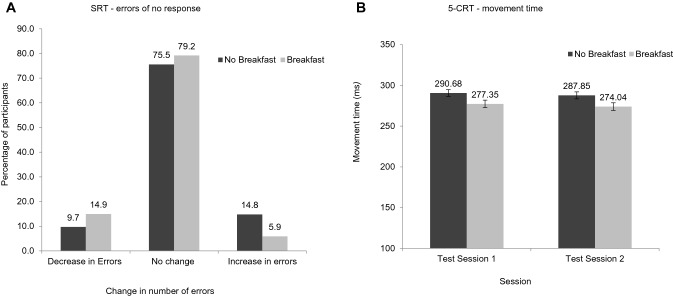
**a** Percentage of participants who made fewer, more, or no change in the number of errors of no response on SRT relative to baseline pooled across test sessions one and two. *SRT* Simple Reaction time. **b** Least Squares Mean ± SE movement time (ms) for 5-CRT according to intervention and test session. *5-CRT* 5 choice reaction time

A significant effect of the intervention was shown for SRT accuracy (CMH[1] = 8.67, *p* < 0.01). A larger proportion of participants increased the number of errors of no response they made across the morning relative to baseline in the no breakfast intervention (14.8%) compared with the breakfast intervention (5.9%; Fig. 3a).

For 5-CRT movement time (ms), the distribution of residuals showed a positive skew and was normalised by the removal of eleven outliers. The final ANCOVA model for 5-CRT movement time (ms) demonstrated a significant main effect of the intervention (*F*[1,204] = 9.90, *p* < 0.01) and a significant baseline*session interaction (*F*[1,203] = 12.75, *p* < 0.001). LSMeans comparisons indicated that at mean baseline performance the difference between interventions was significant (*t*[204] = 3.15, *p* < 0.01; Table 4). As shown in Fig. 3b, movement time was faster following breakfast vs. no breakfast at test sessions one and two. There were no significant effects of the intervention on all other SRT and 5-CRT outcome variables.

#### Visual-sustained attention

The distribution of residuals for the number of correct targets for blocks 3 and 4 showed a negative skew and was normalised by the removal of three outliers. The analysis demonstrated a significant main effect of the intervention for Block 3 (*F*[1,202] = 6.00, *p* < 0.05), a significant baseline*intervention interaction for Block 3 and 4 (Block 3: *F*[1,202] = 6.29, *p* < 0.05; Block 4: *F*[1,202] = 4.01, *p* < 0.05), and a significant baseline*session interaction for Block 4 (*F*[1,202] = 4.54, *p* < 0.05). The LSMeans comparison indicated no difference between interventions at mean baseline for Block 3 and 4 across test sessions one and two (Block 3: mean baseline 7.44; *t*(202) = 0.02 ns; Block 4: mean baseline 7.42; *t*(202) = 1.25 ns; Table 4). However, for block 3, the LSMeans comparison between interventions was only significant when baseline = 0 (*t*[202] = − 2.45, *p* < 0.05), when baseline = 2 (*t*[202] = − 2.41, *p* < 0.05) and when baseline = 10 (*t*[202]2.16, *p* < 0.05). For block 4, the LSMeans comparison between interventions was only significant when baseline = 9 (*t*[202] = 2.30, *p* < 0.05). Performance across test sessions one and two was better following breakfast vs. no breakfast in participants with low performance at baseline only in block three. Conversely, performance across test sessions one and two was better following no breakfast vs. breakfast in participants with high performance at baseline only in block three and four.

The distribution of residuals for RVIP false alarms showed a positive skew and was normalised by the removal of eight outliers. Analysis of RVIP false alarms showed a significant main effect of the intervention (*F*[1,202] = 3.92, *p* < 0.05) and a significant baseline*intervention interaction (*F*[1,202] = 8.19, *p* < 0.01). The LSMeans comparison indicated no difference between interventions when baseline = 5.71 across test sessions one and two (*t*(202) = − 0.00 ns; Table 4). However, the LSMeans comparison between interventions was significant when baseline = 20 (*t*[202] = 2.58, *p* < 0.05), when baseline = 50 (*t*[202] = 2.82, *p* < 0.01) and when baseline = 0 (*t*[202] − 1.98, *p* < 0.05). The advantage for breakfast was evident only for participants with high baseline values (i.e. poorer baseline performance) across test sessions one and two.

The residuals for guessing tendency (*B*″) showed a negative skew and were normalised by the removal of 14 outliers. For guessing tendency (*B*″), the analysis demonstrated a significant main effect of the intervention (*F*[1,218] = 10.24, *p* < 0.01) and a baseline*intervention interaction (*F*[1,218] = 9.74, *p* < 0.01). LSMeans comparison when baseline = 0.84 (mean baseline) and when baseline = 1.00 did not confirm a significant difference between the interventions overall across test sessions one and two (*t*[218] = − 0.76 ns and *t*[218] = 1.95 ns respectively; Table 4). However, LSMeans comparison between interventions when baseline = 0.20 was significant (*t*[202] = − 3.21, *p* < 0.01). The interaction was driven by lower levels of baseline, such that the beneficial effect of breakfast across test sessions one and two was evident for those with a poorer performance at baseline only. There were no significant effects of the intervention on all other RVIP outcome variables.

### Secondary outcomes: subjective state and subjective cognitive test performance

The analysis of ratings of perceived hunger and energy levels showed a similar pattern of results to each other. For subjective ratings of perceived hunger and energy levels, the ANCOVAs demonstrated a main effect of the intervention (smallest *F*[1,212] = 54.13, *p* < 0.0001) and significant intervention*time (smallest *F*[5,1130] = 2.54, *p* < 0.05) interactions. Significant baseline*intervention*time (*F*[5,1130] = 4.66, *p* < 0.001) and baseline*time (*F*[5,1130] = 4.39, *p* < 0.001) interactions were also demonstrated for hunger ratings and a baseline*intervention (*F*[1,212] = 19.85, *p* < 0.0001) interaction for energy ratings. LSMeans comparisons between the interventions at each of the time points indicated that hunger ratings were significantly higher and energy ratings lower in the no breakfast intervention at T3, T4, T5, T6 and T7 (largest *p* = 0.0082; Table 5).

**Table 5:** VAS ratings of subjective state by intervention and test session

VAS descriptor	Baseline (T1)^b^	Baseline (T2)^b^	Post-intervention (T3)	Pre TS one (T4)	Post TS one (T5)	Pre TS two (T6)	Post TS two (T7)
Hunger							
Breakfast	66.70 ± 2.90	63.42 ± 2.89	19.86 ± 2.33	40.96 ± 2.99	38.30 ± 3.06	75.91 ± 2.45	77.53 ± 2.48
No breakfast	66.02 ± 2.88	64.54 ± 2.60	72.46 ± 2.84	72.81 ± 2.85	74.62 ± 2.80	82.53 ± 2.38	85.49 ± 2.08
*p* value^a^	–	–	< 0.0001	< 0.0001	< 0.0001	0.0042	0.0016
Cheerfulness							
Breakfast	51.34 ± 2.96	51.78 ± 2.76	76.35 ± 2.41	65.84 ± 2.83	62.52 ± 2.96	62.40 ± 3.09	55.31 ± 3.25
No breakfast	55.01 ± 2.66	53.30 ± 2.68	50.08 ± 3.08	49.41 ± 3.15	49.76 ± 3.15	55.57 ± 3.21	56.96 ± 3.22
*p* value^a^	–	–	< 0.0001	< 0.0001	0.0017	0.06	0.73
Bad temper							
Breakfast	26.99 ± 2.41	28.88 ± 2.55	17.21 ± 2.23	23.60 ± 2.70	23.06 ± 2.85	26.24 ± 2.85	28.59 ± 3.10
No breakfast	23.24 ± 2.50	23.58 ± 2.51	30.98 ± 3.14	26.61 ± 2.79	32.70 ± 3.29	24.68 ± 2.83	25.61 ± 3.12
*p* value^a^	–	–	< 0.0001	0.20	0.0008	0.89	0.96
Energy							
Breakfast	44.59 ± 2.66	44.34 ± 2.77	76.49 ± 2.21	70.12 ± 2.48	69.14 ± 2.61	58.92 ± 3.04	52.28 ± 3.06
No breakfast	45.77 ± 2.31	45.59 ± 2.45	40.29 ± 2.91	41.57 ± 2.85	41.79 ± 2.89	40.84 ± 2.89	40.66 ± 3.05
*p* value^a^	–	–	< 0.0001	< 0.0001	< 0.0001	< 0.0001	0.0082
Keenness to try hard							
Breakfast	63.34 ± 2.50	62.42 ± 2.75	74.72 ± 2.42	67.85 ± 2.66	69.68 ± 2.69	67.03 ± 2.89	62.80 ± 3.07
No breakfast	63.77 ± 2.61	61.00 ± 2.61	54.06 ± 3.03	58.26 ± 2.97	55.17 ± 3.11	62.54 ± 3.05	61.86 ± 3.09
*p* value^a^	–	–	< 0.0001	0.0067	< 0.0001	0.23	0.65
Distractibility							
Breakfast	45.27 ± 2.98	41.25 ± 3.00	36.13 ± 3.13	41.22 ± 3.04	38.13 ± 3.17	43.64 ± 3.17	48.49 ± 3.43
No breakfast	45.21 ± 2.76	40.20 ± 2.96	50.72 ± 3.17	49.01 ± 3.29	46.30 ± 3.31	46.58 ± 3.36	46.31 ± 3.41
*p* value^a^	–	–	0.0002	0.02	0.01	0.20	0.65
Ease of focus							
Breakfast	63.03 ± 2.65	59.29 ± 2.75	74.10 ± 2.59	73.21 ± 2.46	71.00 ± 2.73	65.53 ± 2.92	65.02 ± 3.07
No breakfast	65.09 ± 2.69	60.78 ± 2.77	57.71 ± 2.90	62.35 ± 2.95	61.98 ± 2.93	64.66 ± 2.88	62.51 ± 3.01
*p* value^a^	–	–	< 0.0001	0.0006	0.0012	0.69	0.20
Awake							
Breakfast	48.37 ± 3.00	49.84 ± 2.96	78.15 ± 2.30	74.43 ± 2.59	71.79 ± 2.78	69.94 ± 2.82	68.85 ± 3.07
No breakfast	49.05 ± 2.78	47.10 ± 2.80	51.87 ± 3.21	55.88 ± 3.31	50.70 ± 3.32	62.57 ± 3.25	61.26 ± 3.26
*p* value^a^	–	–	< 0.0001	< 0.0001	< 0.0001	0.23	0.11

Values are LSmeans ± SEs unless otherwise indicated. T1–T7 corresponds to test day schedule (see Table 2)

*T* time point, *TS* test session VAS, *VAS* Visual Analogue Scale

^a^*p* values are LSMeans comparisons between interventions at T3-T7

^b^Values are means ± SEs. Baseline VAS ratings were as a covariate in the analysis

The analysis of perceived cheerfulness, keenness to try hard, ease of distractibility, ease of focussing, and feeling awake showed a similar pattern of results to each other. For ratings of perceived cheerfulness, keenness to try hard, perceived ease of distractibility, perceived ease of focussing, and ratings of feeling awake, the ANCOVA models demonstrated significant main effects of the intervention [smallest (*F*[1,212] = 3.92, *p* < 0.05)]. Furthermore, significant intervention*time interactions were demonstrated for perceived cheerfulness, keenness to try hard and ratings of feeling awake [smallest *F*[5,1129] = 2.59, *p* < 0.05)] and significant baseline*intervention interactions for ratings of perceived ease of distractibility and perceived ease of focussing (smallest *F*[1,212] = 15.05, *p* < 0.0001). A significant baseline*time (*F*[5,1128] = 4.13, *p* < 0.001) interaction was demonstrated for ratings of feeling awake and a significant baseline*intervention*time (*F*[5,1129] = 2.56, *p* < 0.05) interaction for perceived ease of distractibility. LSMeans comparisons between the interventions at each of the time points indicated that participants who consumed breakfast felt more keen to try hard, able to focus, awake, and less distractible than those in the no breakfast intervention at T3, T4 and T5 (largest *p* = 0.02; Table 5).

For ratings of perceived bad temper, the ANCOVA demonstrated a significant baseline*intervention*time interaction (*F*[5,1127] = 3.69, *p* < 0.01) and a significant main effect of the intervention (*F*[1,212] = 7.26, *p* < 0.01). LSMeans comparisons between the interventions at each of the time points indicated that those who skipped breakfast felt significantly more bad tempered immediately post breakfast (T3, *p* < 0.0001) and immediately following Test Session 1 (T5, *p* < 0.001) than those who had eaten breakfast (Table 5).

For ratings of perceived concentration during the cognitive test battery, the ANCOVA revealed a significant main effect of the intervention (*F*[1,211] = 7.83, *p* < 0.01). LSMeans comparisons between the interventions at the two post-intervention test sessions indicated that participants in the breakfast intervention reported concentrating significantly more than those in the no breakfast intervention at Test Session 1 (*p* < 0.01) (Table 6). The ANCOVA models revealed no significant effects of the intervention on perceived performance, ratings of frustration during the testing battery, and perceived test battery difficultly and, therefore, the LSMeans comparison were not consulted.

**Table 6:** VAS ratings of subjective cognitive test performance by intervention and test session

VAS descriptor	Baseline (T2)^b^	Post TS one (T5)	Post TS two (T7)
Perceived difficulty			
Breakfast	33.84 ± 2.47	31.38 ± 2.75	38.61 ± 3.00
No breakfast	38.40 ± 2.43	35.93 ± 2.76	36.37 ± 3.07
*p* value^a^	–	0.54	0.32
Perceived level of concentration			
Breakfast	71.86 ± 2.22	75.80 ± 2.26	72.45 ± 2.46
No breakfast	74.57 ± 2.10	69.35 ± 2.65	70.68 ± 2.71
*p* value^a^	–	0.0012	0.33
Perceived performance			
Breakfast	65.56 ± 2.26	71.40 ± 2.29	68.19 ± 2.73
No breakfast	62.52 ± 2.31	66.29 ± 2.63	66.13 ± 2.77
*p* value^a^	–	0.46	0.98
Perceived frustration			
Breakfast	37.96 ± 2.50	39.57 ± 3.08	49.09 ± 3.44
No breakfast	37.34 ± 2.58	43.53 ± 3.00	40.32 ± 3.35
*p* value^a^	–	0.30	0.01

Values are LSmeans ± SEs unless otherwise indicated. T2, T5, and T7 corresponds to test day schedule (see Table 2)

*T* time point, *TS* test session *VAS* Visual Analogue Scale

^a^*p* values are LSMeans comparisons between interventions at T5 and T7

^b^Values are means ± SEs. Baseline VAS ratings were included as a covariate in the analysis

## Discussion

### Principal findings

The findings of this study demonstrated that breakfast consumption vs. no breakfast has a positive acute effect on cognitive function and subjective state in 11–13-year-olds. This study employed a randomised controlled trial design and recruited one of the largest samples of adolescents reported in the literature to date. Furthermore, the study used a battery of cognitive tests with previously demonstrated sensitivity to similar acute nutritional manipulations to ensure null findings are due to true lack of effect rather than test insensitivity [17]. The study extends previous research by providing new evidence under highly ecologically valid research conditions by including a school-based testing environment alongside the normal school day and an ad libitum breakfast served in a naturalistic school breakfast programme environment.

There was a positive effect of breakfast on each of the cognitive tasks included in the battery, which measured reaction time, visual-sustained attention and visual-spatial memory. The functions assessed have some wider impact on learning in the classroom. Measures of cognitive performance provide a proxy for cognitive abilities such as the ability to concentrate, react and remember, all of which are key processes for effective learning in school [17, 35, 36]. The positive effects of breakfast consumption on the study’s primary outcome (visual-spatial memory) suggest that breakfast may help children learn at school and could improve academic attainment. Previous studies have demonstrated that visual-spatial memory is a predictor of children’s academic performance [26–28]. The findings suggest that breakfast omission may be associated with poorer cognitive performance on domains that impact negatively on everyday functioning at school. However, the clinical significance of the results is unclear. Pham and Hasson [26] examined the association between visuospatial working memory and reading ability in a sample of schoolchildren. The inclusion of visuospatial working memory into a hierarchical regression model provided significant results, contributing an additional 4% of unique variance to reading comprehension. Whilst Pham and Hasson’s findings [26] suggest that the positive effects of breakfast consumption on the current study’s primary outcome (visual-spatial memory) may have a clinical significant effect on reading ability, our study used different tests of visual-spatial memory and statistical analyses. Therefore, it would be tenuous to directly translate our findings into changes to academic performance.

The results from the reaction time tasks indicate that reaction time was significantly faster following breakfast compared with no breakfast. Both reaction time (faster psychomotor speed) and accuracy were improved by breakfast consumption. Breakfast-induced improvements in reaction time have been previously reported in adolescents, suggesting that this finding is reliable [3, 23]. Notably, in a similar study to the current study, Cooper et al. [3] conducted a school-based, randomised controlled, crossover study comparing the effects of consuming an ad-libitum breakfast relative to fasting in 40 healthy British adolescents aged 12–15 years. The results demonstrated that accuracy on SRT was superior + 20 min post-breakfast consumption vs. fasting [3].

The results of this study also show an advantage for breakfast on visual-sustained attention, evidenced by a significantly greater number of correct responses in Blocks 3 and 4, fewer false alarms, and less guessing. In our previous systematic reviews, we reported that tasks that required attention were facilitated most consistently by breakfast consumption relative to fasting [12, 13]. In a similar study to the current study, Wesnes et al. [23] demonstrated that the consumption of a 45 g portion of RTEC with milk for breakfast relative to fasting reduced the decline in ‘Power of Attention’ factor scores, which included response times on digit vigilance, across the morning in 9–16-year-olds [23]. Taken together, the findings indicate that breakfast consumption facilitates adolescents’ ability to sustain attention across time and the ability to pick out salient information and ignore irrelevant information.

Visual-spatial memory was better following breakfast compared with no breakfast in the current study. Participants in the breakfast intervention were able to recall the locations of a greater number of stimuli. More children who ate breakfast progressed to the highest, most difficult level of the task and made fewer errors compared to those who skipped breakfast. An advantage for breakfast on visual-spatial memory has been demonstrated in previous studies in adolescents [23, 37].

There were several indications that the effects of breakfast on cognitive performance differed according to cognitive performance at baseline, rarely examined in previous studies of healthy well-nourished adolescents. The interaction of intervention with baseline cognitive performance indicated a greater advantage for breakfast when baseline performance was poorer. Similarly, when IQ scores were included as a covariate, consumption of breakfast benefitted those with a lower IQ to a greater extent [38, 39]. Furthermore, previous studies have shown that the positive effects of breakfast consumption relative to fasting tended to be more consistent in undernourished children (typically defined as below-normal height or weight for age). These children also performed more poorly on the cognitive tasks [40–42] and therefore had greater scope for improvement. This demonstrates the importance of the choice of the cognitive task such that floor effects in undernourished participants and ceiling effects particularly in well-nourished adolescents are avoided. This also highlights the importance of sampling so that adolescents with a broad range of cognitive ability are included rather than those at the upper end of the distribution whose cognitive reserve is likely to protect them from the detrimental effects of breakfast omission [38, 39].

Clear positive effects of breakfast consumption were demonstrated on subjective VAS ratings of hunger, mood, motivation, and alertness. Furthermore, these effects were apparent immediately after consuming breakfast and continued until the mid- or late-morning. These findings concur with previous findings demonstrating consistent advantageous effects on subjective feelings of mood, motivation and alertness following breakfast consumption relative to no breakfast in adolescents [3, 4, 37]. Subjective state, such as mood, is an important outcome in its own right, but mood can influence cognitive function [43–45]. Breakfast may affect cognition indirectly through changes in feelings or subjective state (e.g., mood or alertness). The positive changes in mood, alertness, and motivation after breakfast may facilitate cognitive function by increasing the ability to concentrate and/or motivation to try hard on cognitive tasks. There is evidence that mood state modulates cognitive function, but the nature of the relationship is not straightforward. Studies in adolescents have shown that mood and cognitive performance are related, but the nature of the relationship differs before and after cognitive testing. Before cognitive testing, ratings of ‘happy’, ‘friendly’, ‘relaxed’, ‘calm’, ‘angry’, ‘sad’ and ‘dissatisfied’ are negatively associated with thhheee cognitive performance [46, 47]. Feeling more nervous before the cognitive testing is positively associated with thhhe cognitive performance [46]. After cognitive testing, feelings such as ‘friendly’,’ calm’, ‘happy’, ‘contented’ are negatively associated with cognitive performance. Feelings such as ‘drowsy’, ‘sluggish’, ‘tired’ are positively associated with performance [46, 47]. The unexpected finding that feeling more friendly and happy is associated with poorer performance may be because these adolescents feel more relaxed and friendly towards the researchers and are, therefore, not motivated or aroused by the testing situation. Similarly, adolescents who felt more nervous before the cognitive testing may have performed better because they were more aroused by the testing situation which in turn enhanced their attention and response. Negative feelings such as ‘sluggish’, ‘drowsy’ after the cognitive testing may have been associated with superior performance because these participants tried harder or were more engaged with the cognitive tasks and so were feeling more fatigued after trying to perform well. However, studies in children and adolescents have shown that acute improvements in subjective feelings of mood, motivation and alertness are not always accompanied by improvements in cognitive performance [4, 48] which suggests other mechanisms may facilitate cognitive performance.

This sample of adolescents consisted of a mixed sample of habitual breakfast consumers and non-breakfast consumers. It was deemed important to establish if any differences in habitual breakfast behaviour existed across study breakfast interventions and include this variable as a covariate as this would be likely to influence the effects of breakfast consumption, and breakfast omission, on cognitive performance and subjective state. For example, habitual breakfast consumers are likely to be accustomed to regular breakfast and, therefore, fasting may have more adversely affected cognitive performance and subjective state relative to non-breakfast consumers.

There are several possible mechanisms of action for the observed acute cognitive effects of breakfast consumption. These include increased brain glucose availability, glucose-mediated insulin delivery to the brain, increased acetylcholine synthesis, and amplification of the cortisol response. These potential mechanisms have been discussed in detail in our previous systematic reviews [12, 13].

### Limitations

The limitations of this study should be considered when interpreting the findings. The school testing environment is a key strength in terms of ecological validity, but also a limitation. This trade-off between experimental control and ecological validity caused a significant loss of control over the study procedures and extraneous variables. Similarly, there was a trade-off between the ecological validity provided by the ad-libitum breakfast manipulation and the variability in intake between participants which was not controlled. In this situation, the four RTECs did not provide matched macro- or micronutrients, but the RTECs were all high carbohydrate and reasonably matched. Moreover, this study only compared one type of breakfast (RTEC and milk) vs. no breakfast. Hence, as a breakfast vs. no breakfast comparison, the results of this study are not able indicate the optimal breakfast composition for cognitive function. However, other breakfast types have also demonstrated positive effects on cognition in adolescents [12, 13]. This may have influenced the results of the study. For example, within the limited number of studies comparing breakfast type, there is some evidence that suggests that lower glycaemic index (GI) or glycaemic load (GL) breakfasts may facilitate cognitive function relative to higher-GI or GL breakfasts [13]. This suggests that the lower GL/GI RTEC breakfasts included in the current study’s breakfast intervention, that elicit a glycaemic response characterised by less oscillating glucose concentrations and a sustained blood glucose concentration above fasting concentrations, may have facilitated cognitive function to a greater extent. However, the previous evidence is not consistent [15, 49]. Furthermore, simultaneous blood glucose measures are not always taken in studies that reported an advantage of lower-GI or -GL breakfasts on cognition [21, 23, 50]. Moreover, in studies that used continuous blood glucose monitoring, the evidence indicated that large differences in postprandial glycaemic responses elicited by high- and low- GL breakfast interventions were apparent in the absence of any cognitive performance effects [49]. Additionally, there is evidence that positive cognitive effects are apparent when postprandial blood glucose concentrations had returned to baseline [13]. These temporal relations suggest that other factors associated with ingestion of these low-GI breakfast meals, rather than glucose response per se, may mediate the effects on cognitive performance [15].

A major limitation of employing a breakfast vs. no breakfast comparison is the inherent inability to blind participants to the study interventions. The potential bias caused by the inability to blind participants to treatment interventions is exacerbated in a repeated measures design because it increases expectancy effects due to increased familiarity with the study procedures and intervention. Hence, the use of a parallel groups design was preferred for the current study. However, it is likely that this design introduced additional variation between interventions. Furthermore, the effects of breakfast on actual academic performance and the chronic effects of consuming breakfast were not examined. Therefore, it is not possible to confidently conclude that acute changes in cognitive performance will translate to changes in academic performance over time. Nonetheless, the present study adds to an increasing body of literature suggesting the benefits of regular breakfast intake for academic performance [8].

Breakfast cereals are a commonly consumed breakfast food in British adolescents and, therefore, offers good ecologically validity for the sample under study [7, 20]. However, we acknowledge that this type of breakfast may not be generalizable to European adolescents breakfast consumption habits [51]. Finally, statistical correction for multiple testing of the secondary outcomes was not conducted and hence the probability of obtaining significant results will have increased merely because of the number of comparisons. Therefore, the results of the secondary outcomes analyses should be considered exploratory.

### Implications

The findings from this ecologically valid school-based study could have implications for school food provision, such as school breakfast clubs and programmes. Breakfast clubs may offer an avenue by which to increase breakfast consumption by providing an opportunity to eat breakfast immediately before school with peers. Schools also have an important role to play as they present a setting to provide healthy food at breakfast and apply healthy eating messages as part of the curriculum. Moreover, a review of the benefits of school breakfast clubs reported that breakfast clubs offer benefits to cognitive and academic performance and social development, which may be more pronounced in breakfast clubs operating in deprived areas [52]. Encouragingly, many schools have school breakfast programmes, but the availability is greater in primary than secondary schools [20]. Hence, the findings of this study suggest that adolescents also represent an important target population for promoting breakfast consumption, possibly via the provision of breakfast clubs, which may benefit cognitive function and learning at school. Furthermore, the findings highlight the need for national school food policy to consider the universal provision of school breakfast, particularly in adolescents.

Another area of work which requires attention is the acute effect of breakfast composition on cognitive performance. Previously systematic reviews have demonstrated a shortage of studies and problematic designs [12, 13]. Further studies are needed with well-matched study interventions to establish the role of breakfast composition in schoolchildren’s cognitive performance. This may help make feasible recommendations on the type of breakfast that is beneficial for cognitive performance in schoolchildren to serve in school breakfast programme environments.

## Conclusion

To conclude, breakfast consumption has a positive acute effect on subjective state, attention, reaction time, and memory in adolescents. These findings have important implications because adolescents often skip breakfast on school days. Moreover, these findings have important implications because breakfast consumption represents a modifiable lifestyle factor which could be manipulated to enhance the learning of children and adolescents. Efforts that encourage the regular consumption of breakfast on school days (e.g. provision of free school breakfasts) are, therefore warranted.
